# In vivo and in vitro models show unexpected degrees of virulence among *Toxoplasma gondii* type II and III isolates from sheep

**DOI:** 10.1186/s13567-021-00953-7

**Published:** 2021-06-10

**Authors:** Mercedes Fernández-Escobar, Rafael Calero-Bernal, Javier Regidor-Cerrillo, Raquel Vallejo, Julio Benavides, Esther Collantes-Fernández, Luis Miguel Ortega-Mora

**Affiliations:** 1 SALUVET, Animal Health Department, Faculty of Veterinary Sciences, Complutense University of Madrid, Madrid, Spain; 2 SALUVET-Innova S.L., Faculty of Veterinary Sciences, Complutense University of Madrid, Madrid, Spain; 3 Mountain Livestock Institute (CSIC-ULE), León, Spain

**Keywords:** *Toxoplasma gondii*, Genotype, Murine model, Ovine trophoblast, Virulence degree, Virulence factors

## Abstract

*Toxoplasma gondii* is an important zoonotic agent with high genetic diversity, complex epidemiology, and variable clinical outcomes in animals and humans. In veterinary medicine, this apicomplexan parasite is considered one of the main infectious agents responsible for reproductive failure in small ruminants worldwide. The aim of this study was to phenotypically characterize 10 Spanish *T. gondii* isolates recently obtained from sheep in a normalized mouse model and in an ovine trophoblast cell line (AH-1) as infection target cells. The panel of isolates met selection criteria regarding such parameters as genetic diversity [types II (ToxoDB #1 and #3) and III (#2)], geographical location, and sample of origin (aborted foetal brain tissues or adult sheep myocardium). Evaluations of in vivo mortality, morbidity, parasite burden and histopathology were performed. Important variations between isolates were observed, although all isolates were classified as “nonvirulent” (< 30% cumulative mortality). The isolates TgShSp16 (#3) and TgShSp24 (#2) presented higher degrees of virulence. Significant differences were found in terms of in vitro invasion rates and tachyzoite yield at 72 h post-inoculation (hpi) between TgShSp1 and TgShSp24 isolates, which exhibited the lowest and highest rates, respectively. The study of the *CS3*, *ROP18* and *ROP5* loci allelic profiles revealed only type III alleles in ToxoDB #2 isolates and type II alleles in the #1 and #3 isolates included. We concluded that there are relevant intra- and inter-genotype virulence differences in Spanish *T. gondii* isolates, which could not be inferred by genetic characterization using currently described molecular markers.

**Supplementary Information:**

The online version contains supplementary material available at 10.1186/s13567-021-00953-7.

## Introduction

The cosmopolitan apicomplexan parasite *Toxoplasma gondii* can infect almost all homoeothermic species [1]. It is estimated that approximately one third of the global human population is infected by this obligate intracellular protist, and its high prevalence values in primary livestock species support its consideration as an important risk to food safety [2]. *Toxoplasma gondii* infection is commonly subclinical in immunocompetent individuals; however, it may cause important disorders in immunocompromised and pregnant hosts [1]. In this regard, *T. gondii* is one of the main causes of reproductive failure in small ruminants and is responsible for approximately 10% of ovine abortions in Europe [3], thereby implying notable economic losses caused to the sheep industry worldwide.

*Toxoplasma gondii* possesses significant genetic and phenotypic diversity that has been proposed to be responsible for variations in clinical presentation. Initially, the *T. gondii* global population was assumed to be structured in three clonal lineages associated to virulence in a murine model (highly virulent type I, moderately virulent type II, and nonvirulent type III) [4–7]. Nonetheless, the studies were strongly biased by the fact that isolates included were mainly from human patients and domestic animals originating from France and the USA, along with the implementation of monolocus typing strategies promoting the misidentification of atypical and recombinant strains circulating [8]. Two decades of scientific effort in isolation and both molecular and phenotypic characterization of the parasite led to the discovery of a much more complex reality involving a population structure with at least 16 haplogroups worldwide [9–11] and a virulence degree classification under debate [12]. Regarding strains that circulate in European and Spanish sheep livestock, there are several genotyping studies that demonstrate the total predominance of type II strains, which coexist with small percentages of type III and recombinant strains [13–15].

In vivo murine models have been traditionally used to evaluate the virulence degree of *Toxoplasma* isolates by calculating the cumulative mortality rate [16]. On the other hand, in vitro culture models have also been shown to be highly suitable and informative for phenotypic characterization of apicomplexan parasite strains [17–20]. Regarding *T. gondii*, studies employing host target-cell lines such as those derived from central nervous system or placental tissues [21–24] are of special interest. However, most studies have been carried out with laboratory-adapted isolates that are nonrepresentative of *Toxoplasma* population-wide biological diversity.

There is growing evidence of different outcomes when the same strains infect different hosts [25–27]. In this sense, alternative virulence approaches, including allelic combination characterization of demonstrated virulence factors such as *ROP18* and *ROP5* [28–31] or virulence molecular markers such as *CS3* [32], have been tested recently, producing promising results concerning allelic variation linked to virulence.

The present study aimed to characterize the virulence degree of a panel of *T. gondii* isolates recently obtained from naturally infected Spanish sheep through an in vivo murine model (including cumulative mortality and morbidity rates, parasite burdens and pathological lesions evaluation), along with in vitro invasion and proliferation assays in an ovine trophoblast cell line (AH-1). In addition, *CS3*, *ROP18* and *ROP5* allelic profile characterization of all isolates was carried out.

## Materials and methods

### Ethic statement

Animal procedures for the *T. gondii* strains virulence degree evaluation in mice (PROEX 274/16) were approved by the Animal Welfare Committee of the Community of Madrid, Spain, following proceedings described in Spanish and EU regulations (Law 32/2007, R.D. 53/2013, and Council Directive 2010/63/EU). All animals used in this study were handled in strict accordance with good clinical practices, and all efforts were made to minimize suffering. As a humane endpoint, mice with a severe loss of body condition or nervous clinical signs were euthanized to limit unnecessary suffering.

### Mice

Seven-week-old female Swiss/CD1 mice were obtained from a commercial supplier (Janvier Labs, Le Genest-Saint-Isle, France). The animals were free from common viral, parasitic, and bacterial pathogens according to the results of routine screening analyses performed by the manufacturer. Mice were housed with ad libitum access to food and water in a controlled environment with 12-h light and 12-h dark cycles, and the experimental procedures were performed at 8 weeks of age.

### Parasites and cell cultures

A panel of 10 *T. gondii* isolates previously obtained from sheep [15] was selected for phenotypic characterization according to three criteria: (a) genetic diversity, limited to the three predominant PCR–RFLP genotypes present in Spain (ToxoDB #1, #2 or #3); (b) geographical location within Spanish territory; and (c) clinical sample of origin (abortion-derived tissues or myocardial tissues from chronically infected adult sheep) (Table 1). The *T. gondii* isolates included in this study were subjected to a restricted number (from 8 to 12) of lytic cycles completed in cell culture or passages, to preserve their in vivo biological behaviour and avoid adaptation to the cell culture. Parasites were maintained by serial passages in Vero cells (ATCC CCL-81). Briefly, cells were cultured in DMEM (Gibco, Thermo Fisher Scientific, Waltham, MA, USA) supplemented with foetal bovine serum (FBS) (Gibco), penicillin (100 U/mL), streptomycin (100 μg/mL) and amphotericin B (Lonza Group, Basel, Switzerland), at 37 °C and 5% CO_2_ in 75 or 25 cm^2^ tissue culture flasks. Tachyzoites used for in vivo and in vitro assays were recovered from cultures of Vero cells at low passages, when the majority of the parasites were still intracellular, and purified by filtration through a 3-μm polycarbonate filter (IpPORE^®^, IT4IP, Louvain-la-Neuve, Belgium) as previously described [16]. The quantity and viability of tachyzoites were determined by Trypan blue exclusion, followed by direct counting in a Neubauer chamber.

**Table 1:** **Panel of 10** ***Toxoplasma gondii*** **Spanish ovine isolates** [15] **selected for the in vitro and/or in vivo assays on the basis of PCR-RFLP genotype (ToxoDB #1, #2 or #3), geographic origin, and original clinical sample (ovine foetal brain or adult ovine myocardium)**

Isolate ID	Genotype # (ToxoDB)	Geographic origin	Original clinical sample
TgShSp1	#3	Palencia, central Spain	Ovine foetal brain
TgShSp2	#1	Navarra, northern Spain	Ovine foetal brain
TgShSp3	#3	Palencia, central Spain	Ovine foetal brain
TgShSp7	#3	Segovia, central Spain	Ovine foetal brain
TgShSp8	#3	Valencia, eastern Spain	Ovine foetal brain
TgShSp10	#3	Teruel, central Spain	Ovine foetal brain
TgShSp11	#3	Cáceres, western Spain	Adult ovine myocardium
TgShSp16	#3	Badajoz, western Spain	Adult ovine myocardium
TgShSp24	#2	Ciudad Real, central Spain	Adult ovine myocardium
TgShSp30	#3	Badajoz, western Spain	Adult ovine myocardium

For in vitro assays, an immortalized trophoblast cell line (AH-1) originally obtained from primary cultures of ovine placenta was used [33]. The cell line was kindly supplied by the Department of Veterinary Microbiology and Pathology of Washington State University (Pullman, WA, USA). AH-1 cells were cultured under conditions similar to those used for Vero cells.

### Assays of virulence in mice

Two in vivo experiments were conducted to evaluate cumulative mortality and morbidity rates at 42 days post-inoculation (dpi) (Section “Assay A”) and the parasite burden and histological lesions shown by the isolates in mouse organs during the acute and chronic stages of the infection (Section “Assay B”).

#### Assay A

Serial dilutions in phosphate buffered saline (PBS) were performed to obtain doses from 10^5^ to 1 tachyzoite(s) of each isolate per 200 μL. Each dose was intraperitoneally (IP) inoculated into five 8-week-old female Swiss/CD1 mice. Five control mice were inoculated with 200 μL of PBS. Mice were monitored twice daily for 6 weeks, and clinical signs were recorded. The cumulative morbidity rate was evaluated by establishing clinical sign scoring adapted from reference [34]. The cumulative mortality rate was calculated based on the ratio of casualties to the total number of infected mice [16]. Serum samples from mice that were humanely euthanized, presented sudden death, or reached the end of the experiment at 6 weeks post-inoculation were collected and stored at −20 °C until serological procedures for *T. gondii* antibody detection using an indirect fluorescent antibody test (IFAT) to confirm infection.

#### Assay B

An additional group of 10 mice per isolate was IP-inoculated with 10^3^ tachyzoites. Five animals were sacrificed at 7 dpi, and the remaining five mice were sacrificed at 30 dpi to study the acute and chronic phases of the infection, respectively. Selected organs were collected during necropsies for *T. gondii* DNA detection and quantification. Briefly, the mice were bled, and the right cerebral hemisphere, the right eye, the right lung, half of the heart, a piece of a liver lobe, and the right kidney from each mouse were transferred immediately following euthanasia into clean 1.5 mL tubes and stored at −80 °C until DNA extraction. The left cerebral hemisphere, the left lung, half of the heart, the left kidney, and a piece of a liver lobe, the tongue, and the *quadriceps femoris* muscle were fixed in 10% buffered formalin and processed for conventional histological examination. After staining with haematoxylin/eosin, lesions in the samples were subjectively categorized from 0 (no lesion) to 4 (the most severe grade within observed lesions). Serum samples were also collected and stored at −20 °C until analysis.

*Toxoplasma gondii* antibody detection by IFAT and DNA extraction, detection and quantification in assay B were implemented exactly as in reference [12]. In both assays A and B, mouse *Toxoplasma* infections were confirmed by IFAT (titer > 1:25) or by brain or lung tissue imprints.

### In vitro assays in ovine trophoblast target cells

A limited number of six isolates (TgShSp1, TgShSp2, TgShSp3, TgShSp11, TgShSp16 and TgShSp24) were selected for in vitro phenotypic characterization according to the same criteria used in the in vivo experiments but also considering the more contrasting in vivo results. Phenotypic characterization was carried out in AH-1 ovine trophoblast cells [33] since trophoblasts are target cells in congenital *Toxoplasma* infection and mediate the innate immune response [35].

#### Invasion assays

Parasite invasion rate (pInvR) determination was attempted as described previously [36]. Briefly, AH-1 cells were seeded at 1 × 10^5^ cells per well into 24-well culture plates. In total, 200 purified tachyzoites were added per well. Cultures were washed three times with PBS at different time points (4 or 8 hpi) to remove unadhered/non-invading tachyzoites. Unwashed cultures were also included in the study. All plates were fixed for 15 min with cold methanol at 56 hpi and the number of infection events (parasitophorous vacuoles or lysis plaques) per well was determined by applying single immunofluorescence staining (see Section “Immunofluorescence staining”) directly over the wells and counting by direct observation under an inverted fluorescence microscope. Experiments were assayed in triplicate, and three independent experiments were carried out.

The parasite invasion rate at 4 and 8 hpi and the total parasite invasion rate at 56 hpi (pInvR_4h_, pInvR_8h_, and pInvR_T_, respectively) were determined as the number of infection events observed per well in cell monolayers washed at the different time points (4 hpi, 8 hpi or unwashed) divided by two to estimate the percentage of invading tachyzoites. This assay allowed us to determine the time at which each isolate reached the maximum peak of invasion.

#### Immunofluorescence staining

Fixed cultures were permeabilized using a solution of 0.25% Triton 100X in PBS 3% BSA (Bovine Serum Albumin Fraction V; Roche, Germany) (30 min, 37 °C). After washing with PBS (× 3), wells were treated with 3% BSA in PBS for 30 min at room temperature (RT) to block nonspecific antibody binding. Then, parasites were stained using positive murine serum samples from previous experimental infections (1:200 in a 0.3% BSA/PBS solution) [12, 15] as a primary antibody (1 h, 37 °C) and a 1:1000 dilution (in PBS) of goat anti-mouse IgG conjugated to Alexa Fluor^®^ 488 (green, Thermo Fisher Scientific, Waltham, MA, USA) as a secondary antibody (1 h, RT). The nuclei were stained by washing the cells with a solution of 1:10 000 DAPI (4′,6-diamidino-2-phenylindole dihydrochloride; Invitrogen™) in PBS.

#### Proliferation kinetics assays

The proliferation kinetic of each of the isolates in the AH-1 cell line were determined by quantifying the number of tachyzoites at specific times after inoculation (8, 24, 32, 48, 56, 72, 80 and 96 hpi) by real-time PCR (qPCR). Cells were cultured and infected as indicated above, but in this case, a multiplicity of infection (MOI) of 4 was used. Cultures were washed at 8 hpi (time post-inoculation at which the parasite reached > 50% invasion rate, as determined in the previous experiment) and subsequently maintained at 37 °C in a 5% CO_2_ atmosphere. At the selected time points and after removing the supernatant, samples were collected by adding 150 μL of lysis buffer and 20 μL of proteinase K (Qiagen, Hilden, Germany) to each well, transferred into PCR clean 1.5 mL tubes and frozen at −80 °C prior to DNA extraction. In parallel, replicates of cell cultures grown on coverslips were identically infected and fixed at the same time points selected for DNA sample collection. Fixed cultures were labelled by single immunostaining (as described above for the invasion assays) to microscopically study the proliferation kinetics of all isolates included using a confocal fluorescence inverted microscope (CBM-SO Microscopy Services, Madrid, Spain). Experiments were assayed in triplicate, and three independent experiments were carried out.

#### DNA extraction and qPCR parasite quantification

Genomic DNA was extracted from the collected samples using the DNeasy^®^ Blood and Tissue Kit (Qiagen) according to the manufacturer’s instructions. Parasite quantification was carried out by qPCR using primer pairs for the 529-bp repetitive element of *T. gondii* [37]. DNA samples were adjusted to 20 ng/μL, and reactions were performed in a final volume of 25 μL using GoTaq^®^ qPCR Master Mix (Promega, Alcobendas, Madrid, Spain), 10 pmol of each primer and 100 ng of DNA in an Applied Biosystems 7500 FAST Real-Time PCR System (Applied Biosystems, Foster City, CA, USA). Amplification was performed by a standard protocol (10 min at 95 °C, 40 cycles at 95 °C for 15 s, and 60 °C for 1 min). The number of *T. gondii* tachyzoites was calculated by interpolating the average Ct values on a standard curve equivalent to 1 × 10^5^ − 1 × 10^−1^ tachyzoites generated by tenfold serial dilutions of parasite DNA in a solution of ovine genomic DNA at 20 ng/μL. Parasite proliferation was expressed as the parasite number/ng of DNA. Standard curves for *T. gondii* showed an average slope always close to −3.3 and an R^2^ > 0.98.

#### Proliferation kinetics and tachyzoite yield (TY) determination

The parasite proliferation kinetics of each *T. gondii* isolate were studied by plotting the values of tachyzoites/ng of total DNA reached, which was determined by qPCR, against the specific collection time points. The tachyzoite yield at 72 hpi (TY_72h_) was defined as the average number of tachyzoites/ng DNA quantified by qPCR at that time point for each isolate.

### Molecular analyses of predictive markers for virulence in mice

The *CS3* marker [32] and the virulence factors *ROP18* and *ROP5* [28, 30], which are suggested to have high predictive value for the virulence degree in mice, were studied, aiming to provide a correlation between the allelic profile and unexpected differences in phenotypic features observed. Briefly, the methodology was based on nested PCR-DNA sequencing of each marker and the details are summarized in Additional file 1. DNA samples of strains representative of the three canonical clonal lineages were used as references to note TgRH (type I, ToxoDB #10), TgMe49 (type II, #1), and TgNED (type III, #2).

The yielded amplicons were directly subjected to Sanger sequencing in both directions using the internal primers described in Additional file 1. The sequencing procedures were carried out as shown in reference [15] at the Center for Genomic Technologies of the Complutense University of Madrid (Spain). The resulting sequences were imported, read, edited manually if necessary, and analysed using BioEdit software (version 7.0.5.3) [38]. Necessary alignments were performed using Clustal Omega software [39]. Finally, in silico digestion of each locus sequences by identification of restriction enzyme motifs was conducted by the NEBCutter 2.0 program [40]. Specific restriction enzymes are indicated in Additional file 1.

### Data statistical analysis

One-way ANOVA followed by Tukey’s multiple range tests were employed to compare the parasite burden assessed for each isolate within each organ and the time of infection (7 or 30 dpi) studied. The Kruskal–Wallis test was employed for comparisons among the pInvRs shown for the different isolates within each time point (4, 8, and 56 hpi), among the parasite proliferation values (no of parasites/ng DNA) reached by each isolate at the time points included in the proliferation kinetics experiments and finally used to compare the TY_72h_ reached by each isolate. When statistically significant differences were found with the Kruskal–Wallis test, Dunn’s multiple-comparison test was applied to examine all the possible pairwise comparisons. The significance for these analyses was established at *p* < 0.05. GraphPad Prism 6 v.6.01 (San Diego, CA, USA) software was used to perform all statistical analyses and graphical illustrations.

## Results

### In vivo characterization in a murine model

#### Cumulative mortality and morbidity rates (assay A)

**Figure 1:**
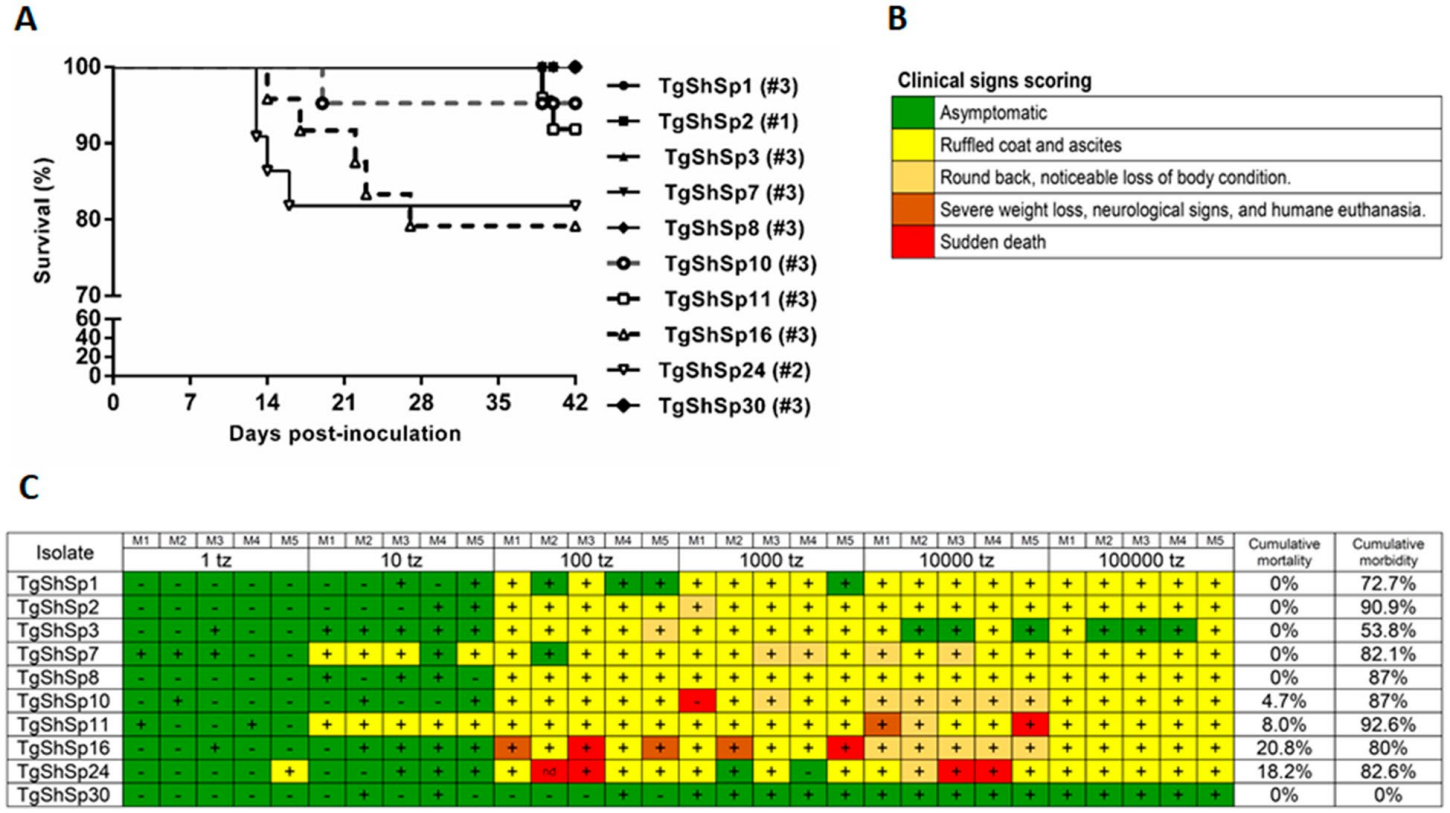
**Cumulative mortality and morbidity parameters in the evaluation of virulence degree in *Toxoplasma gondii* isolates. A** Kaplan–Meier survival curves of *Toxoplasma gondii* isolates from sheep. ToxoDB# genotype of each isolate is indicated. **B** Clinical sign scoring used in the evaluation of morbidity. **C** Summary of the cumulative mortality and morbidity parameters in the evaluation of virulence degree. The gradation of clinical signs observed in mice inoculated with each isolate is shown. Coloured boxes represent the most severe clinical sign observed in each individual mouse challenged with each dose according to the clinical sign scoring included below. + : mice with a *Toxoplasma*-infection confirmed by IFAT or tissue imprints of brain or lung. − : mice without a confirmed *Toxoplasma*-infection. nd: not done. Cumulative mortality and morbidity rates are indicated. The dosages 10^5^, 10^4^,10^3^, 10^2^, and 10^1^ are included for cumulative mortality rate calculation, whereas all dosages were considered for cumulative morbidity rate calculation.

The cumulative mortality rate was 4.7% for TgShSp10, 8.0% for TgShSp11, 20.8% for TgShSp16, 18.2% for TgShSp24, and 0% for the rest of the isolates, as shown in the survival curves (Figure 1A). Therefore, all isolates must be classified as nonvirulent (cumulative mortality < 30%) according to the criteria established in [41]. Regarding the cumulated morbidity rate, the maximum clinical sign score reached in each inoculated mouse is shown in Figures 1B and C. Isolates TgShSp7, TgShSp10, TgShSp11, TgShSp16 and TgShSp24 were noted to cause a rounded back and noticeable loss of body condition in a higher proportion compared to the other isolates, even causing severe weight loss and nervous signs and consequently humane euthanasia in the case of TgShSp11 and TgShSp16. TgShSp16 and TgShSp24 stood out in terms of spontaneous death (2/25 and 4/23, respectively). On the other hand, the TgShSp1, TgShSp3, TgShSp8 and TgShSp30 isolates triggered only mild clinical signs in a lower proportion of infected mice, specifically including a ruffled coat and ascites; besides, the clinical signs completely receded quickly.

#### Tissue tropism, parasite burden and histopathological lesions (assay B)

**Figure 2:**
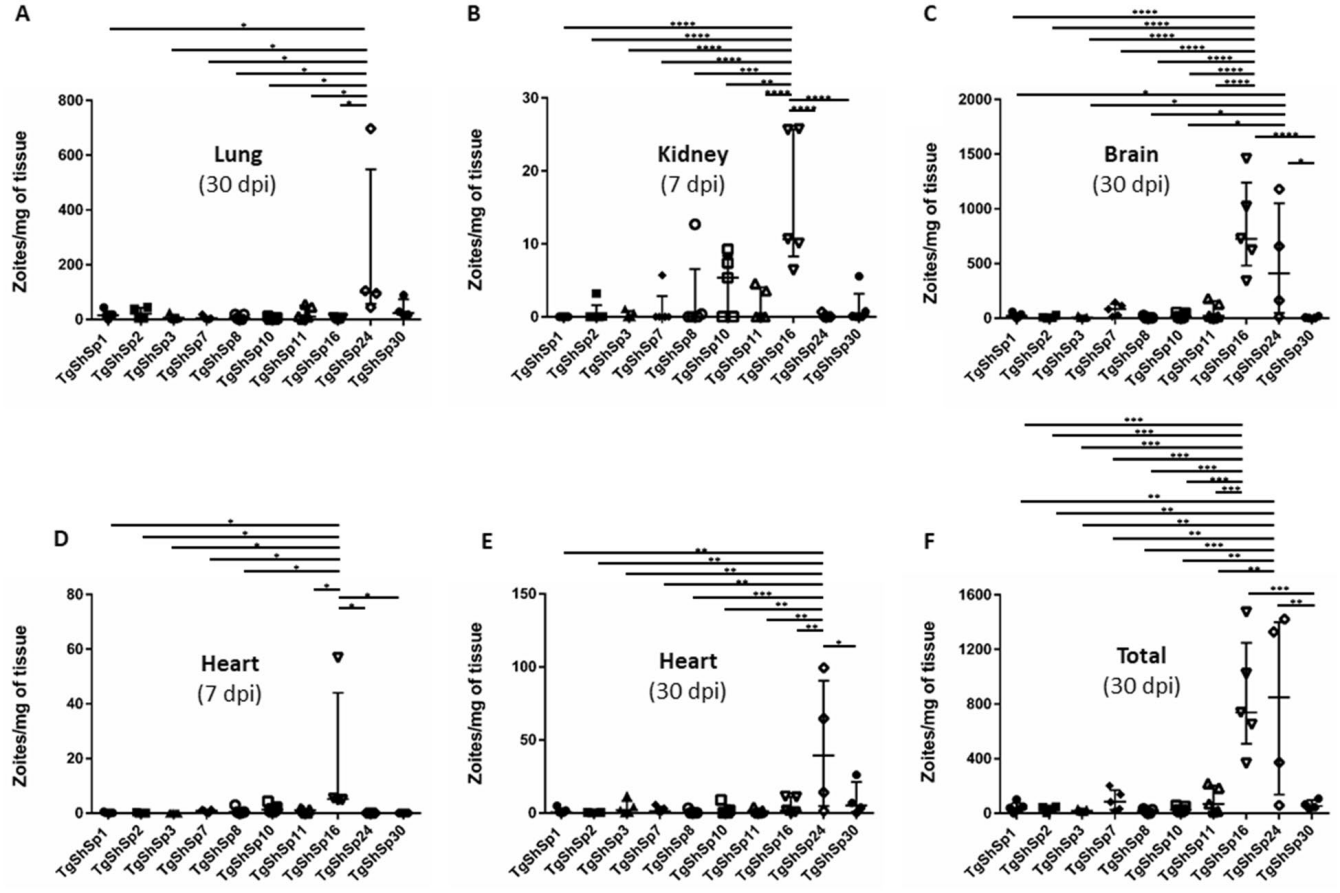
**Parasite loads (zoites/mg of tissue) found in different mouse organs.** **A** Brain at 30 dpi; **B** lung at 30 dpi; **C** heart at 7 dpi; **D** heart at 30 dpi; **E** kidney at 7 dpi; and **F** total parasite burden per mouse at 30 dpi. Median and interquartile values are represented. One-way ANOVA followed by Tukey’s test; *p < 0.05, **p < 0.01, ***p < 0.001, ****p < 0.0001.

The parasite burden reached by each isolate in the different tissues studied is shown in Figure 2. None of the mice scheduled for sacrifice at 7 dpi seroconverted, whereas those scheduled to be sacrificed at 30 dpi all seroconverted except for one mouse in each group infected with the isolates TgShSp2, TgShSp24 and TgShSp30, which in consequence were not considered for the calculation of the parasite loads. In the case of the lung, the parasite burden during the acute phase was low in general (unpublished observations) and higher during the chronic phase for all isolates except TgShSp7, TgShSp10 and TgShSp16, with the TgShSp24 isolate showing the highest mean value (Figure 2A). Regarding the parasite burden in acute tropism tissues such us liver and kidney, values were considered negligible in mice sacrificed at 30 dpi (unpublished observations). For both organs at 7 dpi, all mean parasite burden values were low except those reached by TgShSp16 in kidney tissues with 100% of animals having parasite DNA present (5/5) (Figure 2B). Concerning the brain at 30 dpi (Figure 2C), TgShSp16 and TgShSp24 isolates drew attention for having the highest median values with notably high parasite loads (726.5 and 410.3 zoites/mg of brain tissue, respectively). The mean parasite burden reached in the brain by TgShSp16 was between 10 and 100 times higher than of the rest of the isolates (*p* < 0.0001), and twice that of TgShSp24 (no statistically significant differences; *p* > 0.05). On the other hand, the TgShSp8 and TgShSp30 isolates stood out for having the lowest median parasite loads, closely followed by TgShSp2 and TgShSp3. With respect to heart tissues, all isolates showed a higher mean burden at 30 dpi than at 7 dpi, with the exception of the TgShSp16 isolate, whose parasite load during acute infection was significantly higher than that of the rest of the isolates except TgShSp10 (Figures 2D and E). Notably, the TgShSp24 mean parasite burden reached at 30 dpi was many times the average burden reached by the rest of the strains at this infection stage, supposing statistically significant differences (Figure 2E). Ocular tissue showed great variability in DNA detection and quantification, making it difficult to interpret the results (unpublished observations). However, the TgShSp24 isolate caused 100% of infected animals to exhibit parasite DNA in the eye (4/4) and a median value of 5.6 parasites/mg of tissue on average during the chronic phase. Likewise, strain TgShSp16 resulted in 60% of infected animals with parasite DNA in ocular tissues (3/5) and a median value of 8.2 parasites/mg of tissue at the same time point. Overall, the total parasite burden reached in mice infected with TgShSp16 and TgShSp24 at 30 dpi was significantly higher than that of the rest of the isolates (Figure 2F). On the other hand, TgShSp8 and TgShSp3 produced the lowest total parasite burdens.

**Figure 3:**
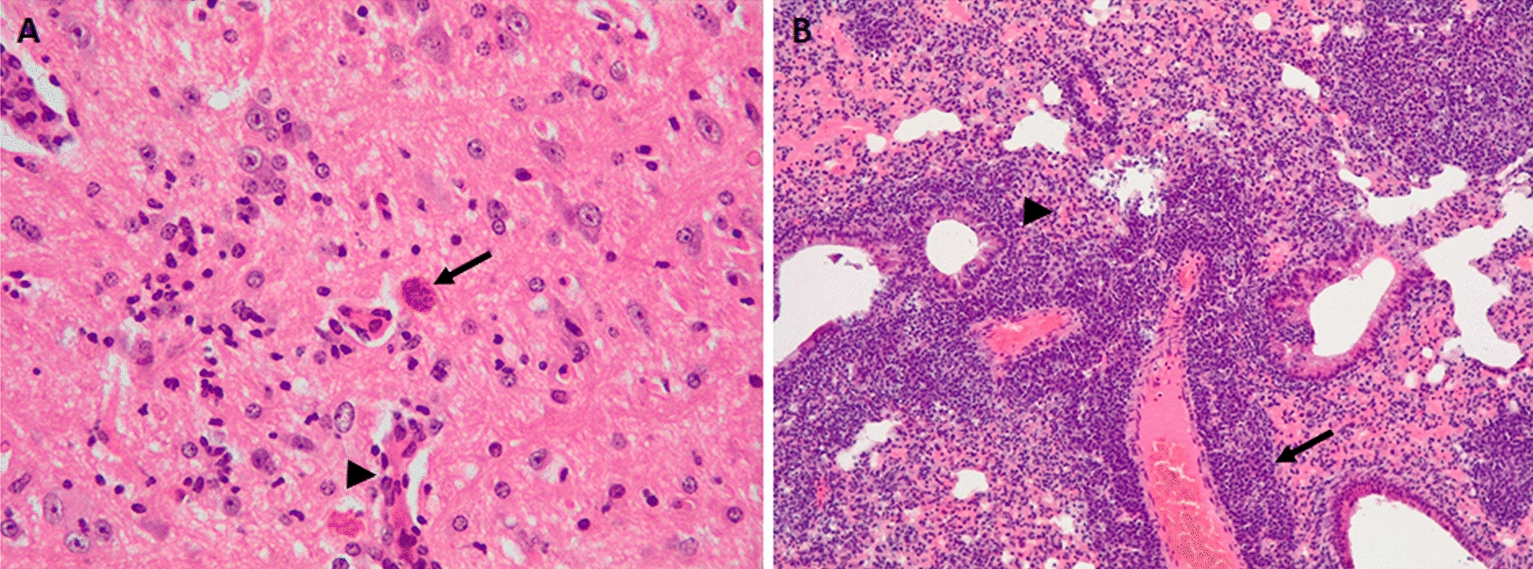
**Histological lesions observed in *Toxoplasma gondii* infected mice. A** Brain. Mouse infected with the TgShSp16 strain. Note, the tissue cyst-like structure (arrow) and non-purulent vasculitis (arrowhead). **B** Lung. Mouse infected with the TgShSp24 strain. Note, the perivascular infiltration of non-purulent inflammatory cells (arrow) and focal thickening of the alveolar wall (arrowhead).

Histological lesions were mainly observed in the brain, liver, and lung, where multifocal aggregates of mononuclear inflammatory cells were detected. The intensity of the inflammatory infiltrate ranged from clusters of scant lymphocytes (grade 1) to aggregates of numerous lymphocytes, macrophages and plasma cells, frequently in relation to blood vessels (grade 4). Lesions were notably more frequent in tissues collected at 30 dpi than at 7 dpi. No lesions were found in the kidney. In the liver, nonspecific inflammatory lesions were observed in mice infected with all isolates studied at 30 dpi, but they were observed more often and with greater severity in the case of mice infected with TgShSp1, TgShSp11 and TgShSp24 isolates. Lesions in the brain were distinguished by glial foci and perivascular infiltration of inflammatory cells mainly present in a chronic phase of the infection. Mice infected by TgShSp16 and TgShSp24 isolates stood out from the rest of mice due to the severity of the brain lesions (especially TgShSp24-infected tissues, which showed a case of grade 4 lesions) and tissue cyst-like structures presence in the unique case of TgShSp16-infected brains (three animals) (Figure 3A). Mice infected with TgShSp2, TgShSp3, and TgShSp30 isolates showed minimal or no presence of brain lesions at 30 dpi. In the case of lung tissues, mice inoculated with the TgShSp24 isolate were highlighted again for presenting increasingly severe foci of inflammation (two animals had grade 3 lesions, and two animals had grade 4 lesions) (Figure 3B).

Histological lesions detected in heart tissues were less abundant, with 0–20% of mice affected in the case of TgShSp1, TgShSp2, TgShSp3, TgShSp7, TgShSp8, TgShSp10, TgShSp11 and TgShSp30 infections, which contrasted with the figures of 60% in the case of TgShSp16 and 80% in the case of TgShSp24-infected mice, noting in even higher severity in the latter case (two animals had grade 1 lesions, and two animals had grade 2 lesions). Portions of quadriceps and tongue tissues, as skeletal muscle instances, were also evaluated for inflammation signs. Lesions in the quadriceps were only found in mice infected with TgShSp7, TgShSp16, TgShSp24 and TgShSp30 isolates at 30 dpi, with those from TgShSp24-inoculated mice highlighted due to much higher significance (one animal had grade 2 lesions, and two animals had grade 3 lesions). Inflammation foci in tongue tissues were even scarcer and were only detected in mice inoculated with TgShSp1, TgShSp7, TgShSp24 y TgShSp30 at 30 dpi, again with more severe degree in the case of TgShSp24-infected animals (two animals had grade 2 lesions). The histopathological evaluation data were in strong agreement with the results of the parasite load quantification.

### *In vitro characterization in an ovine trophoblast target cell*

**Figure 4:**
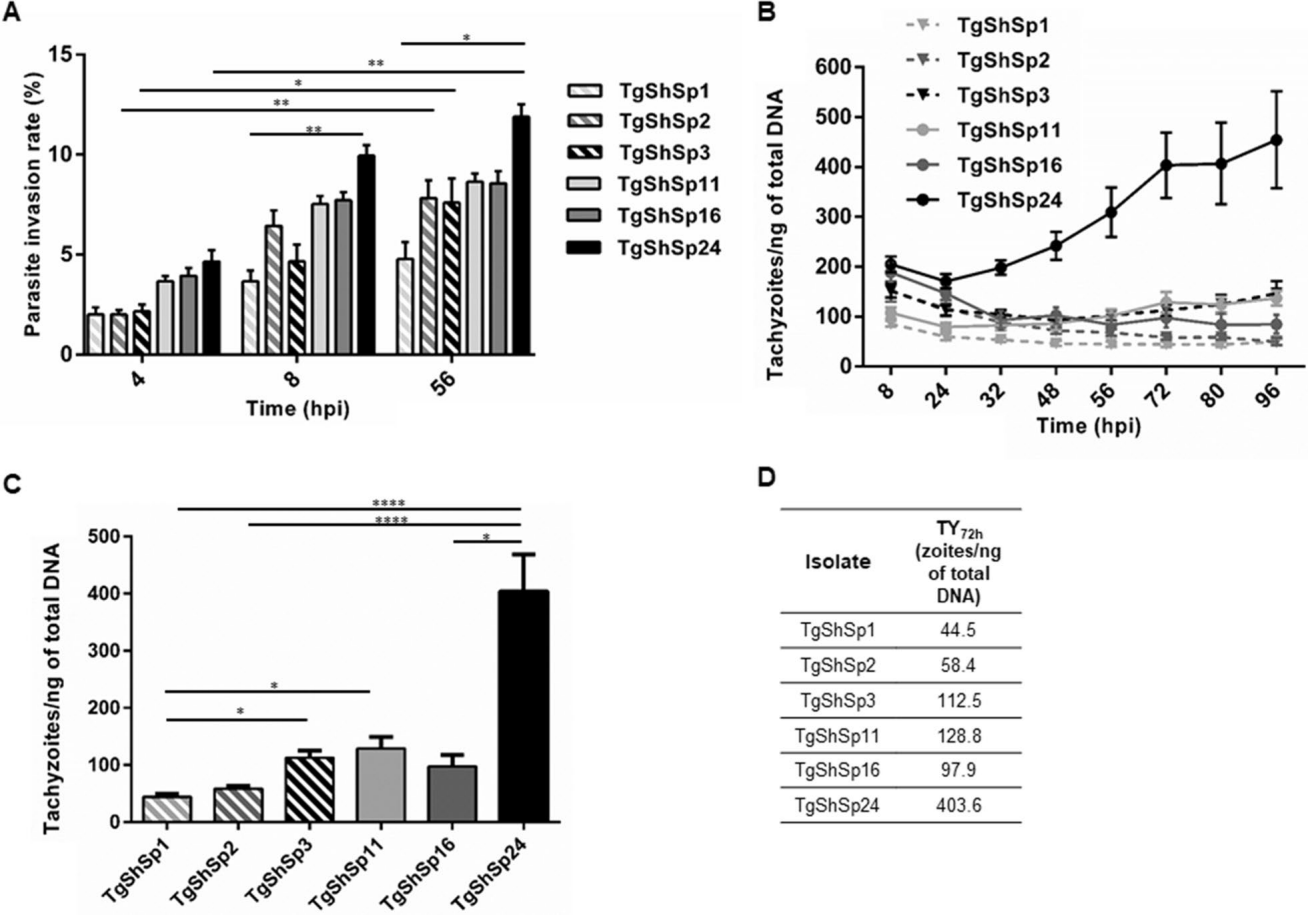
**Differential growth of *Toxoplasma gondii* isolates in ovine trophoblast AH-1 cells. A** Parasite invasion rate at 4 and 8 hpi and total parasite invasion rate at 56 hpi (pInvR_4h_, pInvR_8h_, and pInvR_T_, respectively) of TgShSp1, TgShSp2, TgShSp3, TgShSp11, TgShSp16 and TgShSp24 isolates infecting AH-1 cells. Parasite infection rates were defined as the percentage of invading tachyzoites (number of infection events per well) at the different time points for each isolate. Each column and error bar represent the mean and the SD of 3 replicates from 3 independent assays at the indicated sampling times. **B** Proliferation kinetics of each of the isolates in AH-1 cells. The number of tachyzoites/ng of total DNA reached at specific times after infection (8, 24, 32, 48, 56, 72, 80 and 96 hpi) by each isolate is represented. TgShSp1 vs*.* TgShSp24 at 32 h (*p* < 0.01), 48 h (*p* = 0.0001), 56 h (*p* < 0.0001), 72 h (*p* < 0.0001), 80 h (*p* < 0.0001), and 96 h (*p* < 0.001); TgShSp2 vs*.* TgShSp24 at 56 h (*p* < 0.05), 72 h (*p* < 0.001), 80 h (*p* < 0.01), and 96 h (*p* < 0.001). **C**, **D** Tachyzoite yield at 72 hpi (TY_72h_) reached by each isolate. TY_72h_ mean values for each isolate are tabulated. *p < 0.05; **p < 0.01; ***p < 0.001; ****p < 0.0001.

The parasite invasion rates at 4 and 8 hpi, and the total parasite invasion rate at 56 hpi (pInvR_4h_, pInvR_8h_, and pInvR_T_) of the TgShSp1, TgShSp2, TgShSp3, TgShSp11, TgShSp16 and TgShSp24 isolates in the AH-1 cell line are shown in Figure 4A. The percentage of invading tachyzoites varied significantly between 4 and 56 hpi for TgShSp2, TgShSp3 and TgShSp24 but not for the rest of the isolates tested. TgShSp1 and TgShSp24 were the isolates with lower and higher parasite invasion rates, respectively, with significant differences between them at 8 hpi (3.7% and 10%, respectively; *p* < 0.01) and at 56 hpi (4.8% and 11.9%; *p* < 0.05).

The parasite proliferation kinetics of each *T. gondii* isolate are plotted in Figure 4B. In terms of tachyzoites/ng of total DNA produced, there were significant differences between TgShSp1 and TgShSp24 isolates from 32 to 96 hpi and between TgShSp2 and TgShSp24 from 56 hpi until the end of the experiment. Since 72 hpi appeared to be the point at which the isolate TgShSp24 (the only isolate with growth kinetics that fit the exponential growth equation) completed a lytic cycle, we decided to estimate the tachyzoite yield at that time point (TY_72h_) and compared it between isolates (Figure 4C). Significant differences (*p* < 0.0001) were detected in the number of tachyzoites/ng of total DNA reached at 72 hpi between TgShSp1 and TgShSp24 and between TgShSp2 and TgShSp24. Additionally, slight differences (*p* < 0.05) were found between TgShSp1 and both TgShSp3 and TgShSp11 isolates and finally between TgShSp16 and TgShSp24 (specific x̄ TY_72h_ values for each isolate are presented in Figure 4D).

**Figure 5:**
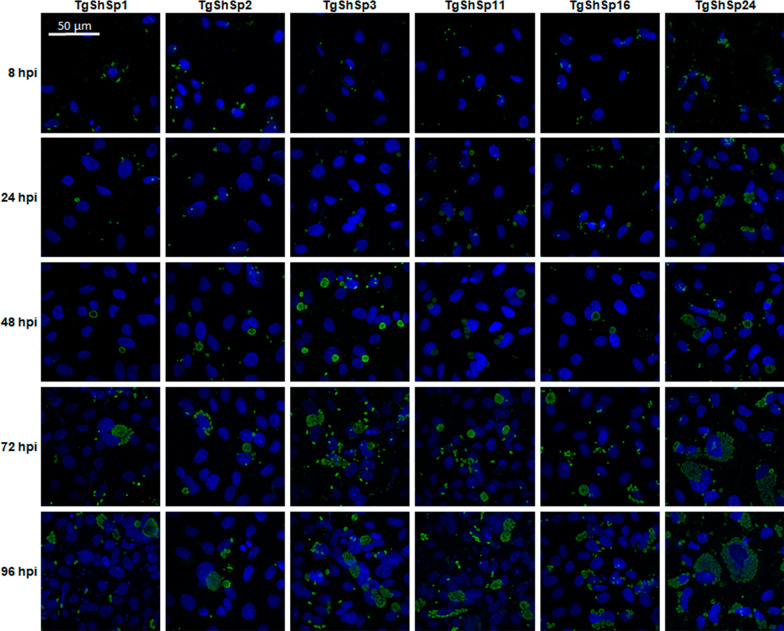
**Proliferation kinetics of *Toxoplasma gondii* isolates determined by microscopic examination of infected cell cultures subjected to immunostaining**. Representative images at 8, 24, 48, 72, and 96 hpi were selected. The scale bar (50 µm) applies to all micrographs.

Microscopic examination of infected cell cultures subjected to immunostaining at different time points showed that the multiplication of the isolates studied began between 8 and 24 hpi (Figure 5). Notable differences in the parasitophorous vacuole sizes between isolates were observed from 24 hpi onwards, with much larger vacuoles in TgShSp24-infected cells than in the rest of the infections. Furthermore, differences in the number of infection events were found between isolates. TgShSp1 and TgShSp2 appeared to successfully infect in a lower proportion than the TgShSp3, TgShSp11 and TgShSp16 isolates. Likewise, the TgShSp24 isolate achieved an even higher number of infections with much faster replication of tachyzoites inside each parasitophorous vacuole. Between 56 and 72 hpi, the rupture of host cells and egress of tachyzoites were observed in all cases (Figure 5).

### Allelic profile characterization of *CS3*, *ROP18* and *ROP5* loci

We amplified, sequenced, and virtually digested the *CS3*, *ROP18* and *ROP5* loci of the 10 isolates included in the assays to examine the possible correlation between the allelic profile and virulence in mice [28, 30, 32]. The *CS3* marker presented a type II allele in all isolates with the ToxoDB #1 or 3 genotype, and the type III allele was only detected in the TgShSp24 isolate (ToxoDB #2). *CS3* sequences from the TgShSp1 and TgShSp24 isolates were deposited in GenBank as instances of each allele detected (MW727456-7). Regarding the *ROP18* gene, the upstream promoter insertion sequence (UPS) of the archetypal type III allele was amplified only in the case of the TgShSp24 isolate (“nonvirulent” allele 3), while the rest of the isolates presented allele 2 (“virulent” allele) according to DEL fragment amplification and sequencing [42]. Similarly, the *ROP5* locus was found to have allele 2 (“nonvirulent” allele) in all type II strains but allele 3 (“virulent” allele) in the TgShSp24 isolate [42]. The *CS3*, *ROP18* and *ROP5* allelic profiles of the 10 isolates included are summarized in Table 2.

**Table 2:** **Genotyping of** ***Toxoplasma gondii*** **isolates with virulence in mice-related loci of** ***CS3***, ***ROP18*** **and** ***ROP5***

Strain ID	ToxoDB#	*CS3*	*RO18*	*ROP5*	Virulence^a^	Cumulative mortality in mice (%)	References
TgRH	10	I	1	1	Vir	100	[1, 15, 43]; this study
TgMe49	1	II	2	2	Int	40	[6, 15, 30]; this study
TgNED	2	III	3	3	Unknown	Unknown	[1, 15, 44]; this study
TgShSp1	3	II	2	2	Non	0	This study
TgShSp2	1	II	2	2	Non	0	This study
TgShSp3	3	II	2	2	Non	0	This study
TgShSp7	3	II	2	2	Non	0	This study
TgShSp8	3	II	2	2	Non	0	This study
TgShSp10	3	II	2	2	Non	4.7	This study
TgShSp11	3	II	2	2	Non	8	This study
TgShSp16	3	II	2	2	Non	20.8	This study
TgShSp24	2	III	3	3	Non	18.2	This study
TgShSp30	3	II	2	2	Non	0	This study

^a^*Toxoplasma gondii* strains are classified according to cumulative mortality in mice into highly virulent (Vir, 100% mortality); intermediately virulent (Int, 99–30%), and nonvirulent (Non, < 30%) [41].

## Discussion

The virulence degree of *Toxoplasma gondii* strains has been conventionally determined according to the cumulative mortality rate in outbred laboratory mice. In this regard, *T. gondii* clonal lineages I, II and III have been traditionally classified as highly virulent (100% lethality, LD_100_ = 1; type I), intermediate virulent (99–30%, LD_50_ ≥ 1000; type II) and nonvirulent (< 30%, LD_50_ > 10^5^; type III) [4, 41]. However, this simplistic criterion may hide important differences not only in lethality but also in the severity of the clinical outcome [45]. Along with cumulative mortality rates, the virulence degree has also been evaluated by morbidity, parasite burdens and pathological lesions detected in different tissues (e.g., central nervous system) [12], and by other nonlethal infection parameters, such as weight loss, anti-*T. gondii* IgG antibodies and haptoglobin levels in serum, cystogenic capacity, or even animal behavioural changes [31, 45–48]. Variations in specific virulence features have already been demonstrated among strains presenting different genotypes, as well as within some belonging to the same genetic type determined by RFLP-based methods [8, 31, 45, 49, 50]. As in the case of the use of laboratory mice for virulence evaluation in a standardized manner (reviewed in [16]) to obtain comparable results, the use of archetypal strains long-term maintained under laboratory conditions is accepted but not representative of the vast biological diversity of the *Toxoplasma* population. It is well known how maintenance under cell culture conditions during successive passages involves strong phenotypic changes in laboratory-adapted strains [6, 26]. In view of the above situation, we present a comprehensive study of the virulence degree in mice of a panel of Spanish *T. gondii* isolates recently obtained from sheep based on lethal and nonlethal parameters. It should be highlighted that the isolates included in the present experiments belong to the most prevalent genotypes in Spanish farm animals [12, 15] and present a low number of cell culture passages, avoiding adaptation to in vitro laboratory conditions.

In the present study, a panel of isolates was subjected to in vivo virulence assays. Of the 10 isolates evaluated, eight had been classified as type II-PRU variants according to 11 RFLP markers, while examples of clonal type II (TgShSp2) and clonal type III (TgShSp24) were also included [15]. All isolates were classified as nonvirulent (cumulative mortality < 30%; LD_50_ > 10^5^) according to the traditional criterion. Here, most of the type II isolates (ToxoDB #1 and 3) presented cumulative mortalities of 0 or close to 0, except TgShSp16, which stood out with a ratio of 21%. This isolate presented the highest mean parasite burden in the brain at 30 dpi, which was between 10 and 100 times higher than that of the rest of the type II isolates (*p* < 0.0001), and it was the only case in which tissue cyst-like structures were found in the brain during histopathological analysis of this tissue. Comparable results were only found in the case of the isolate TgShSp24 (ToxoDB #2), which also presented an almost 20% cumulative mortality and similar parasite load values in the brain. The TgShSp16 and TgShSp24 isolates reached similar total parasite burden values at 30 dpi, ranging between 8 and 55 times higher than those of the rest of the isolates. Concretely, the brain was the organ that most contributed to these differences. Overall, histopathological analysis outlined the enhanced ability to disseminate of both TgShSp16 and TgShSp24 isolates, especially that of TgShSp24. Regarding type II isolates, our results are similar to those reported in reference [31], in which the isolate TgCkStk12 (ToxoDB #1) presented 0% mortality and negligible parasite burdens in mouse tissues, while the Moredun M4 isolate (ToxoDB #3) presented 20% mortality and intermediate parasite burden values in murine tissues evaluated. In another report [45], a group of 16 type II Danish isolates assessed showed how the strains that caused more severe loss in mice bodyweight also induced the highest serum haptoglobin and specific antibodies response in the acute phase of the infection but, likewise, significant differences were found between isolates. Although type II *T. gondii* strains confirmed their low virulence in mice in mortality assays, notable differences in infection dynamics were described between isolates recently obtained from ovine tissues.

Additionally, we used the AH-1 ovine trophoblast cell line, which is a target cell during transplacental *Toxoplasma* invasion in ovine gestation, to study the in vitro invasion rate and proliferation kinetics of selected isolates. This cell line was previously used to demonstrate the role of trophoblasts in the initiation and propagation of placental inflammation during ovine enzootic abortion (*Chlamydia abortus*) [35]. The present results showed significant differences between the clonal type III isolate (TgShSp24) and the other 5 type II isolates (clonal and PRU variant) included. TgShSp24 presented the highest invasion rate in AH-1 cells and reached a tachyzoite production (TY_72h_) nine to three times higher than that of the rest of the isolates. However, between type II isolates, there were also important differences. The outstanding TgShSp1 isolate (ToxoDB #3) had the lowest invasion rate and tachyzoite production, closely followed by TgShSp2 (ToxoDB #1) and TgShSp16 (ToxoDB #3), which had slightly higher invasion rates than TgShSp1 but quite similar low TY_72h_ values. TgShSp3 and TgShSp11 were somewhere between regarding both parameters. Tachyzoite production resulted in clear concordance with microscopic monitoring of the infection, with the TgShSp24 isolate developing notably larger parasitophorous vacuoles and more frequent infection events than the rest of the isolates, and the same evident differences between type II isolates. Numerous previous studies have evaluated the in vitro proliferation kinetics of *Toxoplasma* strains in known infection-target tissues (e.g., central nervous system, muscular, or placental tissues, and immune system cells), normally with the goal of testing a drug treatment or the effect of the disruption of a potential virulence effector, or of studying cellular antiparasitic immune response mechanisms [22, 51–56], almost always involving laboratory strains belonging to type I or II (mostly RH and Me49, respectively). Only a few studies have tested in vitro differential phenotypic characteristics between nonlaboratory strains. A report [57] described similar experiments to those performed herein, evaluating invasion, multiplication and cyst formation rates in an HFF (human foreskin fibroblasts) cell line of a set of four type II strains isolated from human congenital infections. Hence, although there were no relevant differences in terms of multiplication rates, invasion and cyst formation rates varied between isolates included. Another research [58] found differences in terms of in vitro growth in a human acute monocytic leukaemia THP-1 cell line between *T. gondii* type II strains; furthermore, it provided evidence of significantly lower proliferation rates in type II strains than in those belonging to the type III genotype. The present study was pioneering in the use of ovine trophoblast cells for virulence evaluation of recently obtained *T. gondii* isolates from natural sheep infections.

Considering both in vivo and in vitro assays, most type II isolates (ToxoDB #1 and #3) possessed nonvirulent characteristics, except for the TgShSp16 isolate (#3), which showed a 21% cumulative mortality rate and an especially relevant enhanced ability to disseminate in vivo to organs such as the brain, despite low-intermediate in vitro invasion and proliferation rates in AH-1 cells. The type III TgShSp24 isolate presented the most virulent profile among the strains evaluated. This finding contradicts former classifications of *T. gondii* isolates that regarded type III as the least virulent in mice strains among the three major linages [4, 41]. Increasing evidence of this inconsistency can be found in recent literature [12, 25, 30]. In a recent study, a clonal type III isolate obtained from an Iberian domestic pig had nearly 90% mortality in Swiss mice in an identical virulence assay [12]. Similarly, in a Japanese study, 100% lethality in mice inoculated with doses of 10^2^-cyst of a type III isolate obtained from a cat was reported [25]. The apparently broken linkage between virulence and genotype demonstrates the limitations of RFLP-genotyping and the need to investigate new *T. gondii* strain virulence markers.

The *CS3* locus has been described as a highly predictive marker of *T. gondii* strains lethality in mice, with several studies in which Brazilian and Chinese isolates exhibiting high mortality rates (normally above 80%) also presented type I or II alleles for the *CS3* locus, while nonvirulent isolates (mainly 0% mortality) showed type III alleles [32, 59, 60]. Hence, our *CS3* typing results completely disagree with those above-mentioned investigations due to the presence of alleles II among the type II isolates assessed (0–21% mortality) and alleles III in the case of the type III isolate (18%). Contradictory results had been also reported in the literature [12, 61], suggesting the need for further research to unravel the definitive role of the locus in *Toxoplasma* virulence.

As an intracellular pathogen, during infection, *T. gondii* governs the cellular immune response through the mobilization of several virulence factors secreted by different specialized organelles. Concretely, a wide list of GRA, ROP and MIC effectors have been described. Quantitative trait locus (QTL) mapping analyses of the virulence of F1 progeny derived from sexual recombination experiments of representative strains of the three *T. gondii* archetypal genotypes resulted in the identification of ROP18 and ROP5 as key determinants of acute virulence in mice [62, 63]. Previous studies concluded that the allelic combination of *ROP18*/*ROP5* is highly predictive of virulence in mice across globally distributed *T. gondii* isolates [28, 30]. Here, we determined that all type II isolates included in virulence assays presented the *ROP18*/*ROP5* allelic combination of 2/2, regardless of the mortality rate reached. The allelic combination of 2/2 has been associated with 0% lethality in mice, with the exception of laboratory strains Me49 and ARI (40 and 60%, respectively), reflecting the influence of long-term laboratory conditions on parasite behaviour [28, 30]. In addition, the TgShSp24 isolate *ROP18*/*ROP5* allelic combination was 3/3, the most unspecific profile due to its association with levels of mortality strongly varying from 100 to 0% [12, 30, 31, 64]. While there appears to be a correlation between the *ROP18*/*ROP5* allelic combination observed in the isolates evaluated and their virulence degree to some extent, additional genetic factors might be also involved.

Recent investigations [19, 64, 65] also accomplished in vivo and in vitro virulence assays of nonlaboratory *Toxoplasma* strains, along with allelic profile characterization of *ROP18* and *ROP5*, among other relevant loci. The first investigation [64] tested the lethality in Swiss-Webster mice, as well as in vitro growth and plaque formation in Vero cells, of four recombinant strains obtained from different Serbian hosts. Similarly, Japanese researchers [65] carried out lethality assessments in CD1 mice, as well as in vitro invasion and cyst formation assays in HFF cells, of different partly genotyped Japanese strains. Finally, the cumulative mortality and morbidity rates in BALB/c mice, along with the growth rate and spontaneous cyst formation ability in HFF cells and primary mouse peritoneal macrophages, of the recently obtained TgCatJpObi1 isolate (genotype #4) was studied in [19]. Our results are not directly comparable with these studies due to the clonal genetic character of our selected strains and the different methodologies implemented; however, it could be said that the Japanese isolates TgCatJpObi1 (#4) [19] and TgCatJpOk3 (haplogroup 2) [65] are phenotypically similar to most of the Spanish type II isolates, with nonvirulent in vivo and in vitro phenotypes and presenting the same *ROP18*/*ROP5* allelic combination of 2/2.

In this study, we showed some new examples of inter- and intra-genotype phenotypic variation in in vivo and in vitro virulence features between recently obtained isolates. Thus, we were able to demonstrate that current widely used genetic characterization methods are not entirely appropriate to predict virulence of *T. gondii* field isolates, drawing attention to the need to implement genetic tools that may allow us to obtain much more detailed, precise, and complete genetic information (such as whole-genome sequencing methods) which in turn may serve to explain the biological variability found.

## Supplementary Information

**Additional file 1*CS3*, *ROP5* and *ROP18* loci genotyping procedures.** *CS3*, *ROP5* and *ROP18* loci genotyping was based on nested PCR of each locus. First, each locus was individually pre-amplified using the corresponding external primers and subsequently amplified by nested PCR using the internal primer pairs and the external PCR products as DNA template. For *ROP18*, one set of three external primers and two sets of internal primers were used. One set of internal primers aimed to amplify a repetitive sequence (DEL) in the promoters of the archetypal type I and II alleles, and the other to amplify the upstream promoter insertion sequence (UPS) exclusive to the archetypal type III allele. The nested PCR products were subjected to Sanger sequencing in both directions using the internal primers. Finally, in silico digestion of each locus sequences by specific restriction enzymes indicated was conducted by the NEBCutter V2.0 program [40]. No restriction enzyme digestion was required to distinguish alleles of the UPS sequence, as product is only generated for the type III allele. DNA samples of strains representative of the three archetypal lineages were used for comparisons, to note TgRH (type I, ToxoDB #10), TgMe49 (type II, #1), and TgNED (type III, #2).

## Data Availability

Data supporting the conclusions of this study are included within the article and the Additional file 1. The *CS3* sequences generated in the present study were submitted to the GenBank database under the accession numbers MW727456-7. Histological samples are available from the authors upon reasonable request.
