# Supplementary material for: In vivo and in vitro models show unexpected degrees of virulence among Toxoplasma gondii type II and III isolates from sheep
Source: Vet Res. 2021 Jun 10;52:82. doi: 10.1186/s13567-021-00953-7 (PMC8194156; doi:10.1186/s13567-021-00953-7)
Supplement: Supplementary file 1 — Additional file 1CS3, ROP5 and ROP18 loci genotyping procedures. CS3, ROP5 and ROP18 loci genotyping was based on nested PCR of each locus. First, each locus was individually pre-amplified using the corresponding external primers and subsequently amplified by nested PCR using the internal primer pairs and the external PCR products as DNA template. For ROP18, one set of three external primers and two sets of internal primers were used. One set of internal primers aimed to amplify a repetitive sequence (DEL) in the promoters of the archetypal type I and II alleles, and the other to amplify the upstream promoter insertion sequence (UPS) exclusive to the archetypal type III allele. The nested PCR products were subjected to Sanger sequencing in both directions using the internal primers. Finally, in silico digestion of each locus sequences by specific restriction enzymes indicated was conducted by the NEBCutter V2.0 program [40]. No restriction enzyme digestion was required to distinguish alleles of the UPS sequence, as product is only generated for the type III allele. DNA samples of strains representative of the three archetypal lineages were used for comparisons, to note TgRH (type I, ToxoDB #10), TgMe49 (type II, #1), and TgNED (type III, #2). [file 13567_2021_953_MOESM1_ESM.docx]

**Additional file 1: Table S1.** ***CS3*, *ROP5* and *ROP18* loci genotyping procedures.**

| Marker | External primers (5’ - 3’) | Tm (°C) | Internal primers (5’ - 3’) | Tm (°C) | \| NEB Restriction enzymes \| \| --- \| | Reference |
| --- | --- | --- | --- | --- | --- | --- | --- |
| *CS3* | CS3-Fext_GTGTATCTCCGAGGGGGTCT | 55 | CS3-Fint_AGCGGATTTCCAACACTGTC | 60 | N1aIII + MboI | [32] |
|  | CS3-Rext_TGTGACTTCTTCGCATCGAC |  | CS3-Rint_CTGCTGCATTCACAAACTCC |  |  |  |
| *ROP5* | ROP5-Fext_GGACAGACGCAGGCTTTTAC | 55 | ROP5-Fint_TGTGGCAGTTCAGTCTCAGC | 55 | BfaI | [28, 30] |
|  | ROP5-Rext_TCAAACGTCCTGACACTTCG |  | ROP5-Rint_TCGAAGTTGAGGAACCGTCT |  |  |  |
| *ROP18* | ROP18-DelFext_CTCGTCGACCACACAGCTAA  (*) | 56 | ROP18-DelFint_AGTTCCCTTCCCTGGTGTCT ($)  ROP18-DelRint2_CACCGCAAGACAGGCTGTCTTC ($) | 60 | ScrFI + MfeI | [28, 30] |
|  | ROP18-UPSFext_TTTTATCGACATCCCGCTTC  ROP18-UPSRext_GAGTGCTTTCTGTCGCTCCT | 55.2 | ROP18-UPSFint_CACAGCATGAGCTTAAGAGTTG (&)  ROP18-UPSRint2_ACAAACTGGACTGGGGTGAG (&) | 54.5 | No enzyme treatment |  |

(*) ROP18-UPSRext is also used here as reverse primer; primer ROP18-DelRext does not exist.

($) Amplify the ROP18-Del fragment (Type III has no PCR products. Others have PCR products for RFLP analysis).

(&) Amplify the ROP18-UPS fragment (Type I and II have no PCR products. Type III has positive PCR products for RFLP analysis).
